# Individual characteristics moderate listening effort in noisy classrooms

**DOI:** 10.1038/s41598-023-40660-1

**Published:** 2023-08-31

**Authors:** Chiara Visentin, Matteo Pellegatti, Maria Garraffa, Alberto Di Domenico, Nicola Prodi

**Affiliations:** 1 Department of Engineering, University of Ferrara, Via Saragat 1, 44122 Ferrara, Italy; 2 Present Address: Institute for Renewable Energy, Eurac Research, Via A. Volta/A. Volta Straße 13/A, 39100 Bolzano-Bozen, Italy; 3 School of Health Sciences, University of East Anglia, Norwich Research Park, Norwich, Norfolk NR4 7TJ UK; 4 Department of Psychological, Health and Territorial Sciences, University of Chieti-Pescara, Via dei Vestini 31, 66100 Chieti, Italy

**Keywords:** Psychology and behaviour, Acoustics

## Abstract

Comprehending the teacher’s message when other students are chatting is challenging. Even though the sound environment is the same for a whole class, differences in individual performance can be observed, which might depend on a variety of personal factors and their specific interaction with the listening condition. This study was designed to explore the role of individual characteristics (reading comprehension, inhibitory control, noise sensitivity) when primary school children perform a listening comprehension task in the presence of a two-talker masker. The results indicated that this type of noise impairs children’s accuracy, effort, and motivation during the task. Its specific impact depended on the level and was modulated by the child’s characteristics. In particular, reading comprehension was found to support task accuracy, whereas inhibitory control moderated the effect of listening condition on the two measures of listening effort included in the study (response time and self-ratings), even though with a different pattern of association. A moderation effect of noise sensitivity on perceived listening effort was also observed. Understanding the relationship between individual characteristics and classroom sound environment has practical implications for the acoustic design of spaces promoting students’ well-being, and supporting their learning performance.

## Introduction

Comprehending speech when people around us are chatting is a challenging task, that school-age children face daily. Primary school students spend almost 90% of their school day listening to speech in the presence of unwanted and task-irrelevant sounds^1^. Despite the recommended maximum unoccupied level of noise of 35 dB(A)^2^, background noise in primary classes can be as high as 65 dB(A) when children are engaged in individual work, peaking up to 70–77 dB(A) during group work^3^.

Different types of sound stimuli concur in creating classroom background noise. They come from outside the school building (e.g., traffic noise), from outside the classroom (e.g., students’ voices from the corridor) and they are generated directly within the classroom (e.g., heating and ventilation sounds, children’s voices). When the effect of these types of noise was directly compared, classroom noise generated by the children was found to be the most detrimental to speech perception^4,5^, listening comprehension^6,7^, and performance in verbal^8^ and math^9^ tasks. The reason can be traced back to mechanisms of auditory distraction^10^. Depending closely on its spectro-temporal characteristics and informational content, a background noise consisting of one, two, or several competing speakers may evoke domain-specific interference (by competing for the same semantic processes required to complete a verbal task) or attentional capture (by shifting the listener’s attention from the task at hand) or a mixture of both mechanisms^11^.

Students at all levels of education report that sounds from inside the classroom (i.e., speaking classmates) occur more often than sounds coming from outside^12–14^. Primary school children, in particular, report that this type of noise interferes the most with their ability to hear the teacher^14,15^, and perceive it as unpleasant^16^. Notably, primary school children, due to their limited attentional span and the more semi-structured teaching methods will be more talkative and exuberant than older students^17^, thus generating the highest levels of chatter noise^18^. It is worth recalling that the effects of noise on younger children are particularly significant compared to older students and adults, given their still-developing cognitive skills and linguistic resources for disambiguation of speech in noise^19^. Therefore, as in a loop, children in the first grades of instruction, where language learning and development of social skills are of central concern, are the population potentially benefitting the most from a reduction in the internal classroom noise levels but also the one generating the highest levels of such high attentional-capture noise, especially detrimental for verbal learning tasks.

Although research in primary school classrooms has traditionally been devoted to speech perception^20^, the extra load on the basic auditory functions imposed by background noise jeopardizes also higher levels of spoken language processing. There is increasing consensus that ensuring optimal speech perception in the classroom is a necessary condition for listening comprehension, but it alone is not enough^21–23^. Indeed, comprehending a spoken message encompasses more than simply identifying the single words. Comprehension is the process of converting spoken language into meaning within the mind^24^. Listening comprehension includes recall, reasoning, and making inferences on the heard message and it is a crucial ability for students if they are to achieve academic success. Studies focusing on the effect of multi-talker noise on listening comprehension suggest that up to signal-to-noise ratios (SNRs; i.e. the difference between the level of the speech and the level of the background noise) of + 10 dB, children are significantly less able to follow oral instructions^6^, comprehend sentences^7^ and answer questions about short narrative passages^25–27^ in the presence of noise compared to quiet.

The consequences of learning in a challenging acoustic environment are not limited to a drop in task accuracy but also include an increased *listening effort*. Indeed, whereas in ideal conditions speech understanding is effortless and largely automatic, in challenging listening situations (e.g., high background noise, non-native listeners) there is a need for an explicit engagement of the listener’s cognitive resources to understand speech, thus yielding an increased effort. Listening effort was defined as “the deliberate allocation of mental resources to overcome obstacles in goal pursuit when carrying out a task that involves listening”^28^.

The relationship between the cognitive demands of the task and effort is not straightforward, but it is modulated by the listener’s motivation^29^. In a listening task, motivation governs how and how well perceptual and cognitive abilities are used to understand what is been said^30^. Motivation interacts with cognitive demands: the stronger the listener’s motivation, the more willing she/he will be to put effort into the task, even when task demands are high (e.g., high background noise). A recent meta-analysis indicates that motivational factors had a small-to-medium effect on listening effort^31^ and the effect size might vary depending on the specific combination of motivational factors (e.g., financial reward, individual traits) and listening effort measure. It is worth noticing that the vast majority of the literature focuses on the adult population, even though motivation is crucial for students in noisy classrooms to continue listening to the teacher and not disengage or withdraw from the lesson.

During lessons in classrooms, the sound environment can be considered to be similar for the whole class (on the hypothesis of a correct acoustic design of the space). However, children’s performance, reactions to background noise, and perceived listening effort might widely differ among them, giving rise to large inter-individual differences^32^. The conceptual model by Reinten et al*.*^33^ indicates that the effects of sound on human performance are moderated by personal factors (e.g., noise sensitivity, emotional state), and theoretical models of effort (FUEL^28^, ELU^34,35^) suggest that part of the inter-individual variability could be accounted for by differences in individual cognitive abilities and knowledge of the language. Notably, it is still unclear whether the effect of the individual characteristics is constant (i.e., independent of the listening condition and thus subtending listener’s performance whatever the sound environment) or moderated by the sound environment (e.g., a stronger effect of individual factors only under specific listening conditions)^36^.

Concerning linguistic skills, individual language abilities (lexical, grammatical, semantic knowledge, receptive vocabulary) have been found to support children’s performance during speech perception^37^ and spatial listening^38^ in noisy conditions. The effect depends on the cognitive demands of the task, characteristics of the background noise (e.g., stationary, multi-talker), and age^39^. Moreover, proficiency in reading comprehension has been found to positively influence children’s accuracy in listening comprehension in noise^40^ but no interaction was found with the effect of the listening condition (different types of noise presented at the same level) on the task.

Among cognitive abilities, it is suggested that working memory is a key factor in moderating listening task performance but other executive components could also be involved, for instance, updating, switching, and inhibition^41^. Inhibition refers to someone’s ability to suppress automatic responses. Inhibitory control of attention, in particular, enables children to selectively attend and focus on a specific speech stream (the teacher) suppressing attention to other distracters (e.g., voices, looking up at other children rather than focusing on the task)^42^. Semantic processing of speech in a competing talker background is linked with inhibitory control capacities in adults^41^ and it might be in primary school children^43^.

The association of cognitive/linguistic abilities with measures of listening effort has been rarely explored. For the adults, greater cognitive capacities (based on tests of working memory, and inhibition, among others) were found to be associated with decreased listening effort^34^, even though the pattern depended on the specific measure of effort^44^. However, work by Stenbäck et al*.*^45,46^ did not find a significant relationship between inhibitory control and self-reported listening effort (even though, in some listening conditions a significant association was found between working memory capacity and listening effort). Likewise, Brännstrom et al*.*^47^ found that working memory and cognitive flexibility were not related to self-rated effort, but, in contrast, better inhibitory control was related to increase perceived effort (independent of the listening condition). Only two studies examined the association between cognitive functions and listening effort for school-age children^27,48^. Both studies presented the children with a task of narrative passage comprehension, used subjective ratings to assess listening effort, and an overall measure of executive functions (Elithorn Mazes Test) to assess individual cognitive abilities. No correlation was found in a multi-talker babble noise (quiet vs. SNR + 10 dB^27^), whereas a significant association was found in one- or four-talker noise (quiet vs. + 5 dB SNR^48^) that was stronger in noisy than in quiet conditions. The result is in line with literature indicating that, when listening to speech with a limited number of concurrent speakers, children are required to allocate greater cognitive resources to segregate the source and suppress attention towards other competing talkers, thus relying more on selective attention/inhibition capacities^49^.

Besides cognitive and linguistic skills, also internal states may interact with the effect of listening conditions on task performance. In this study we examined the role of noise sensitivity, which is defined as “the internal state of an individual that increases her/his degree of reactivity to noise in general”^50^. When it comes to measuring sensitivity to noise, individuals typically report their own experiences using either single-item scales or questionnaires consisting of multiple items that evaluate subjective reactions to noise across various environments. The strong association between noise sensitivity and annoyance has been well-documented, both for adults^51^ and children^52^. Less research was conducted on the relationship between noise sensitivity and cognitive performance. For adults in open-plan offices, it was shown that noise-sensitive persons are more affected by background speech than non-sensitive individuals, in terms of accuracy, perceived disturbance, and distraction^53^. For university students in open-plan environments, it was found that noise sensitivity moderates the effects of noise on a writing task^54^ but not in a collaborative task^55^. However, to our knowledge, no study has explicitly investigated the association between noise sensitivity and children’s cognitive performance, despite the strong evidence of an association between children’s noise sensitivity and behavior^56^, task-switching and mind-wandering^32^, and effortful control in noise^52^.

This study was designed to explore the role of personal factors when primary school children perform a listening comprehension task in the presence of a two-talker masker. Compared to maskers consisting of a larger number of talkers, the two-talker masker was regarded as more representative of the sonic conditions usually encountered by children when listening to the teacher during frontal lessons^39^.

The following research questions were examined:

(i) Do individual factors (reading comprehension, inhibition, noise sensitivity) moderate the effect of background noise on listening comprehension accuracy and listening effort? To account for the specific influence of the type of measure, the listening effort was assessed both with a behavioral measure (single-task response time) and self-report. Based on previous research with children^48^, we predicted that cognitive abilities^48^ but not literacy skills^40^ would moderate the effect of the listening condition. Based on the study with adults by Francis et al*.*^44^ we also predicted that depending on the measure (RT or self-report), a different pattern of interaction between individual characteristics and listening effort would be found.
(ii) Is it possible to explain a larger portion of the variance in the data by taking into account the impact of individual factors in addition to listening conditions? We predicted that a larger part of data variance would be explained, for all the outcomes employed in the study.
(iii) Do individual characteristics and changes in acoustical conditions affect children’s motivation during the task? We predicted that the listening conditions would affect children’s motivation.

## Results

### Sentence comprehension: accuracy

Averaged accuracy results are presented in Table 1 along with SDs, minima, and maxima. Table 2 shows the results of the full model fitted to the accuracy data of the sentence comprehension task. The statistical analysis indicated a significant main effect of listening condition (χ^2^[1] = 23.83, *p* < 0.001), syntactic complexity (χ^2^[1] = 12.03, *p* < 0.001), and reading comprehension (χ^2^[1] = 15.96, *p* < 0.001). All the other predictors were not significant (all *p*s > 0.11). Results indicate that accuracy scores were significantly higher for the easy compared to the hard listening condition, and for sentences with low compared to high syntactic complexity. Moreover, children with high reading comprehension had a higher proportion of correct responses (M 86%) compared to children with low reading comprehension (M 78.9%).

**Table 1:** Upper panel: proportion of correct responses and response times in the two listening conditions (easy and hard), by syntactic complexity: mean, standard deviation, minimum, and maximum values.

	Syntactic complexity: low				Syntactic complexity: high			
	M	SD	Min	Max	M	SD	Min	Max
Listening condition: easy (SNR + 9 dB)								
Accuracy (%)	91.1	13.6	44.2	100	78.9	18.8	16.7	100
Response times [ms]	3268	947	1043	6658	3855	1607	1009	10,668
Listening condition: hard (SNR + 1 dB)								
Accuracy (%)	85.7	13.9	33.3	100	71.2	19.3	5.4	100
Response times [ms]	3423	1112	1846	8652	4190	1609	1675	9496

	Listening condition: easy (SNR + 9 dB)				Listening condition: hard (SNR + 1 dB)			
	M	SD	Min	Max	M	SD	Min	Max
Listening effort	39.8	32.6	0	100	43.2	31.4	0	100
Motivation	78.2	21.6	35	100	77.2	23.1	0	100

Lower panel: self-reported listening effort and motivation in doing the task in the two listening conditions.

**Table 2:** Generalized linear mixed-effect model for sentence comprehension accuracy (upper part) and response times (lower part).

Predictor	Estimate	Std. error	t-value	*p*-value
Sentence comprehension task accuracy				
(Intercept)	2.00	0.16	12.82	< 0.001 ***
Listening condition [easy]	− 0.33	0.07	− 5.03	< 0.001 ***
Task complexity [high]	0.52	0.14	3.75	< 0.001 ***
Reading comprehension [high]	− 0.35	0.09	-4.16	< 0.001 ***
Inhibition [high]	− 0.06	0.08	-0.79	0.43
Noise sensitivity [high]	− 0.13	0.08	− 1.66	0.10
Listening condition: task complexity	− 0.04	0.05	− 0.75	0.45
Listening condition: reading comprehension	0.08	0.07	1.26	0.21
Listening condition: inhibition	− 0.03	0.06	− 0.49	0.63
Listening condition: noise sensitivity	0.08	0.06	1.38	0.17
Marginal R^2^/Conditional R^2^: 0.105/0.321				
AIC: 2493.3				
Sentence comprehension: response time				
(Intercept)	8.24	0.03	315.97	< 0.001 ***
Listening condition [easy]	0.04	0.01	4.31	< 0.001 ***
Task complexity [high]	− 0.06	0.02	− 3.39	< 0.001 ***
Reading comprehension [high]	0.02	0.02	0.75	0.46
Inhibition [high]	− 0.02	0.02	− 1.12	0.26
Noise sensitivity [high]	0.01	0.02	− 1.12	0.26
Listening condition: complexity	− 0.01	0.01	− 0.57	0.57
Listening condition: reading comprehension	− 0.002	0.01	− 0.22	0.83
Listening condition: inhibition	− 0.02	0.01	− 1.70	0.09
Listening condition: noise sensitivity	− 0.01	0.01	− 0.75	0.45
Marginal R^2^/Conditional R^2^: 0.024 / 0.205				
AIC: 44,517.0				

Estimates are given in the model scale. Reference levels: listening condition Hard, task complexity Low, reading comprehension Low, inhibition Low, noise sensitivity Low.

Significance codes for the *p*-values: ***< 0.001, **< 0.01, *< 0.05.

In the full model, the estimate of standard deviation for random effects was 0.55 for participants and 0.84 for items (sentences), thus indicating that the variability introduced by the sentences was higher than the inter-listener variability. The proportion of variance explained by all the factors (fixed and random) was 32.1%. The statistical model with the same random factor and only the listening condition as a fixed factor explained a similar proportion of data variance (R^2^_c_ = 32.3%).

### Sentence comprehension: response time

Averaged RT results (only correct responses) are presented in Table 1 along with SDs, minima, and maxima. Table 2 shows the results of the full model fitted to data of response time. The statistical analysis indicated a significant main effect of listening condition (χ^2^[1] = 23.58, *p* < 0.001) and task complexity (χ^2^[1] = 5.02, *p* = 0.025) and a significant interaction between listening condition and inhibition (χ^2^[1] = 3.05, *p* = 0.048). All the other predictors were not significant (all *p*s > 0.40).

**Figure 1:**
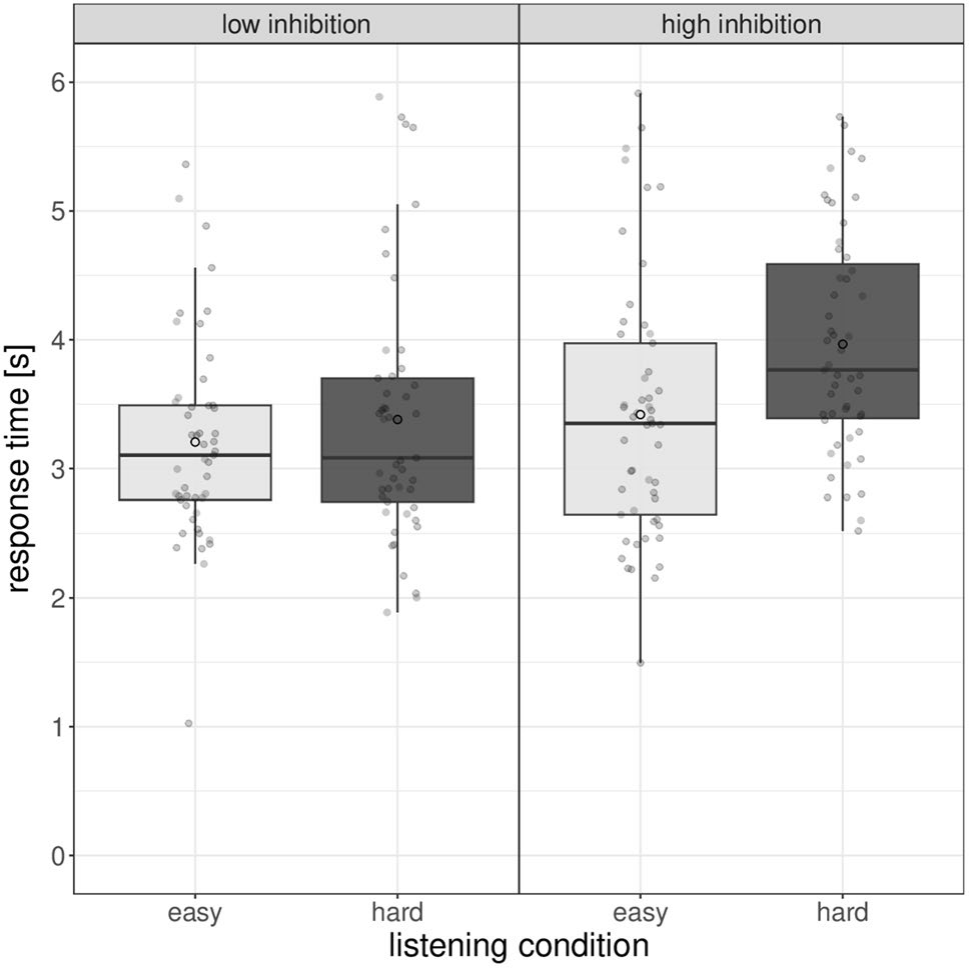
Response times as a function of children’s inhibition and listening condition. Box plots show the median (middle line), mean (white circle) and interquartile range of the data distributions.

Specifically, children had shorter response times in the low compared to the high syntactic complexity condition. The significant interaction is showed in Fig. 1; pairwise tests, with data collapsed across the other variables, indicated that response times for children with low inhibition did not differ significantly between the two listening conditions (easy: M 3321 ms; hard: M 3525 ms; *z* = 1.79, *p* = 0.07), whereas children with high inhibitory control had significantly longer RTs in the hard compared to the easy condition (easy: M 3419 ms; hard: M 4077 ms; *z* = 4.43, *p* < 0.001).

In the full model, the estimate of the standard deviation of the random effect was 0.18 for participants and 0.10 for items, suggesting a larger inter-individual than inter-item variation in RTs. The proportion of variance explained by all the factors (fixed and random) was 20.5%. The statistical model with the same random factors and only the listening condition as a fixed factor explained a smaller proportion of data variance (R^2^_c_ = 19.6%).

### Self-reported listening effort

Averaged self-reported listening effort is presented in Table 1 along with SDs, minima, and maxima. Table 3 shows the results of the full model fitted to the data. The statistical analysis indicated a significant main effect of inhibitory control (χ^2^[1] = 6.73, *p* = 0.009) and a significant interaction between listening condition and noise sensitivity (χ^2^[1] = 4.75, *p* = 0.030). All the other predictors were not significant (all *p*s > 0.12).

**Table 3:** Linear mixed-effect model for the self-rated listening effort (upper part) and motivation (lower part).

Predictor	Estimate	Std. error	t-value	*p*-value
Self-rated listening effort				
(Intercept)	41.49	2.69	15.44	< 0.001 ***
Listening condition [easy]	1.84	1.52	1.21	0.23
Reading comprehension [high]	0.64	2.84	− 0.23	0.82
Inhibition [high]	7.18	2.72	2.64	0.01 **
Noise sensitivity [high]	− 2.79	2.78	− 1.00	0.32
Listening condition: reading comprehension	− 0.75	1.60	− 0.47	0.64
Listening condition: inhibition	− 2.42	1.54	− 1.58	0.12
Listening condition: noise sensitivity	− 3.46	1.57	− 2.21	0.03 **
Marginal R^2^/Conditional R^2^: 0.078/0.554				
AIC: 2001.6				
Self-rated motivation				
(Intercept)	77.93	1.93	40.46	< 0.001 ***
Listening condition [easy]	− 1.08	1.09	− 0.99	0.33
Reading comprehension [high]	− 1.13	2.03	− 0.55	0.58
Noise sensitivity [high]	− 2.41	1.99	− 1.21	0.23
Inhibition [high]	0.81	1.95	0.42	0.68
Listening condition: reading comprehension	2.71	1.15	2.34	0.021 *
Listening condition: noise sensitivity	− 0.15	1.13	− 0.13	0.90
Listening condition: inhibition	− 0.40	1.11	− 0.36	0.72
Marginal R^2^/Conditional R^2^: 0.026 / 0.525				
AIC: 1864.2				

Reference levels: listening condition Hard, reading comprehension Low, inhibition Low, noise sensitivity Low.

Significance codes for the *p*-values: ***< 0.001, **< 0.01, *< 0.05.

**Figure 2:**
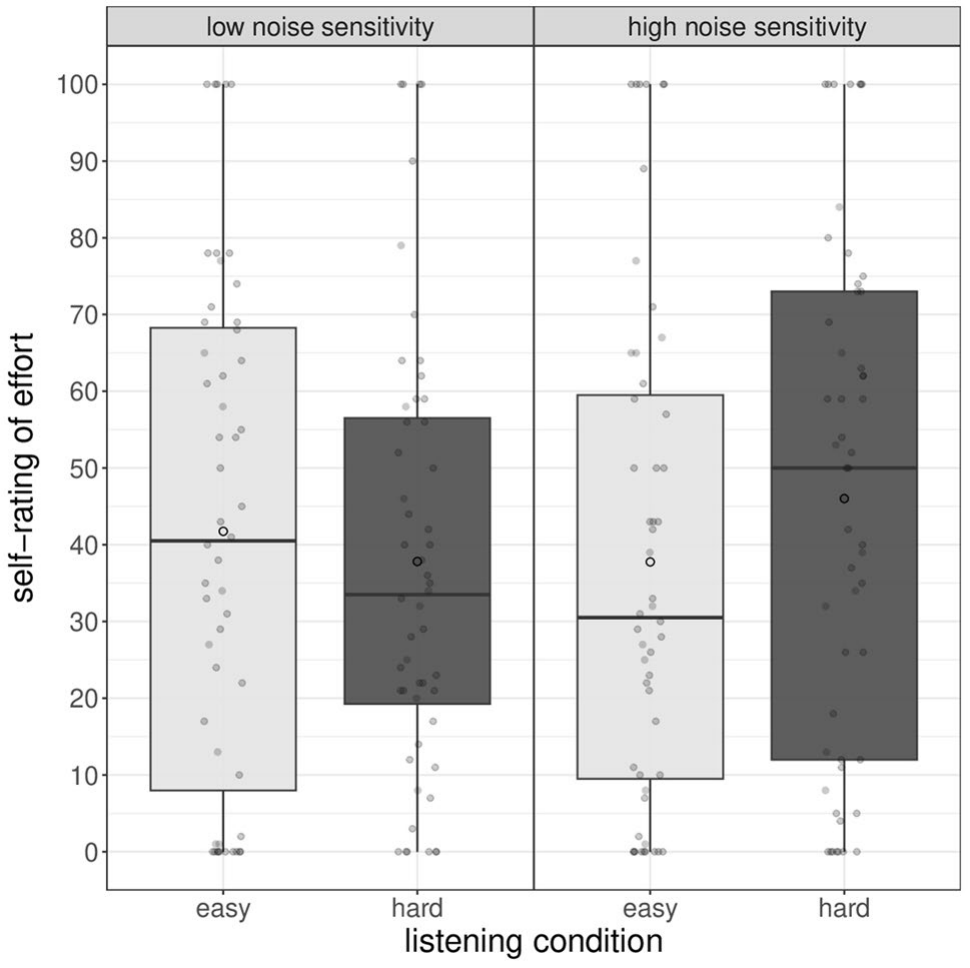
Self-ratings of listening effort as a function of children’s noise sensitivity and listening condition. Box plots show the median (middle line), mean (white circle) and interquartile range of the data distributions.

Specifically, children with a low inhibitory control rated the task as significantly more effortful (M 48.7) than children with high inhibitory control (M 34.3). Figure 2 shows the significant interaction between listening condition and noise sensitivity. Pairwise tests revealed that children with low noise sensitivity rated the two listening conditions as equally effortful (estimated difference = 3.2,* t* = 0.75, *p* = 0.45), whereas children with high noise sensitivity rated the hard condition as significantly more effortful than the easy listening condition (estimated difference = 10.6,* t* = 2.30, *p* = 0.023).

In the full model, the estimate of the standard deviation of the random effect (participant) was 22.0, the residual standard deviation estimate was 21.3, while the proportion of variance explained by all the factors (fixed and random) was 55.4%. The statistical model with the same random factor and only the listening condition as a fixed factor explained a smaller proportion of data variance (R^2^_c_ = 51.4%).

### Self-reported motivation

Averaged self-reported motivation is presented in Table 1 along with SDs, minima, and maxima. Table 3 shows model estimates with associated standard errors and *p*-values. In the model, the estimate of the standard deviation of the random effect (participant) was 15.7 while the residual standard deviation estimate was 15.4. The model intercept was 77.9, thus indicating that children experienced a high level of motivation, at least for the reference levels. The statistical analysis indicated a significant interaction between listening condition and children’s reading comprehension (χ^2^[1] = 5.36, *p* = 0.021). All the other predictors were not significant (all *p*s > 0.23).

**Figure 3:**
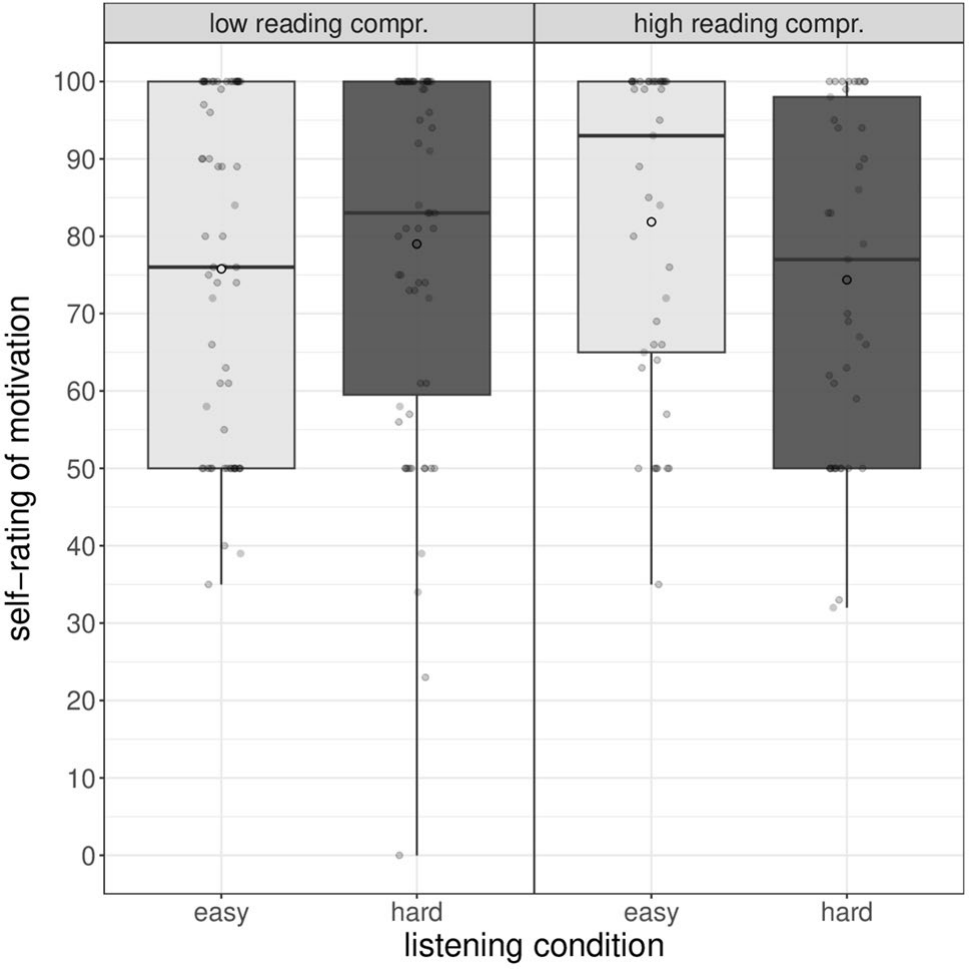
Self-ratings of motivation as a function of children’s reading comprehension and listening condition. Box plots show the median (middle line), mean (white circle) and interquartile range of the data distributions.

The significant interaction is shown in Fig. 3. Pairwise tests indicated that children with low reading comprehension experienced the same level of motivation in both listening conditions (estimated difference = 3.3,* t* = 1.14, *p* = 0.26), whereas children with high reading comprehension felt more motivated in completing the experimental task in the easy then in the hard listening condition (estimated difference = 7.8,* t* = 2.10, *p* = 0.037).

In the full model the proportion of variance explained by all the factors (fixed and random) was 52.5%. The statistical model with the same random factor and only the listening condition as a fixed factor explained a smaller proportion of data variance (R^2^_c_ = 49.7%).

## Discussion

This study aimed to understand whether and how individual factors (reading comprehension, inhibition, noise sensitivity) moderate the effect of listening conditions on accuracy and effort in a listening comprehension task performed by elementary-school children in their classrooms. The dimension of children’s motivation was also investigated, being crucial for active listening in challenging conditions.

The results of the study indicated that children’s performance in the comprehension task improved (better accuracy and shorter RTs) when the level of the two-talker masker was reduced, thus confirming that children significantly benefit from a quiet learning environment not only for speech perception but also when doing complex cognitive tasks.

Children’s reading comprehension had a significant effect on their accuracy in the task, even though no interaction with the listening condition was found. The same pattern (no interaction between listening condition and reading comprehension) was found in a study by Prodi et al*.*^40^, concerning older students performing a sentence comprehension task in more challenging conditions (0 dB SNR) and different types of background noise (classroom noise, traffic noise). Thus, the findings of both studies suggest that listening comprehension is less demanding for children when they are more fluent and proficient in the task^57^ and that having better reading comprehension affords an overall advantage to the children, independent of the listening condition.

Concerning cognitive abilities, we choose to include in the study the inhibitory control, as it supports working memory in solving the confusion between target and competing speech, thus allowing for more resources to be allocated for the processing of the speech content. We found that inhibitory control moderated the effect of the listening condition on the behavioral measure of effort. Only children with better inhibition control experienced an increase in effort (longer RTs) whilst changing from “easy” to “difficult” listening conditions. This may seem counterintuitive. However, the ELU model^34^ allows for the possibility that only listeners with sufficient resources not engaged in the task (e.g., listeners with a high inhibitory control) can experience an increase in effort during acoustically challenging conditions. Research on listening comprehension in noisy conditions for children^48^ and adults^47^ supports the idea that only listeners with better general executive functions or better inhibitory control perceive more effort when the acoustic challenge increases.

The subjective measure of effort included in this study also showed an association with inhibitory control, though with a different pattern than RT, in agreement with the findings by Francis et al*.*^44^. Interestingly, the pattern of our results (greater perceived effort for children with low inhibitory control) is opposite to the study by Brännström et al*.*^47^ even though in both studies no significant interaction was found between this cognitive ability and the acoustic condition. It is possible that the different pattern is related to age differences, as adult listeners participated in the study by Brännström et al*.*^47^. An alternative explanation refers to the background noise used in the two studies (two-talker vs. multi-talker), being that speech perception in a two-talker masker relies more on selective attention skills than in a less informative masker^39^. Further dedicated studies are needed to directly explore the pattern of association between inhibitory control and perceived effort in children, and different types of background noise.

Finally, a moderation effect of noise sensitivity on perceived listening effort was observed, with noise-sensitive children being more affected by high levels of background speech than non-sensitive children. No association between noise sensitivity and task accuracy was found, however. It might be speculated that the quite favorable acoustic conditions of the present experiment were only sufficient to assess the influence of noise sensitivity on perceived effort, and not on accuracy. Additionally, it is important to note that changes in the listening effort do not always mirror those in accuracy scores; in other words, some interventions may affect listening effort while leaving task accuracy untouched. For instance, this is the case of test repetition in noise for primary school students^58^ or the use of a dysphonic voice in a passage comprehension task^26^. To the best of our knowledge, this is the first study investigating the link between noise sensitivity and task performance (in terms of both accuracy and effort) for primary school children, and suggesting that noise-sensitive children might benefit more from improvements in the classroom acoustic environment. Future research could explore further this relationship with reference to different, real-world scenarios.

Results revealed a significant effect of the syntactic complexity of the task, with greater task difficulty being associated with lower accuracy and longer RTs for correct responses. The effect of syntactic complexity is robust and confirms a well know trend in language acquisition of more complex sentences to require more resources and developing later^59^. The effect observed in our study was independent of the listening condition. Previous studies on adults (see for instance^60^) and older children (11–13 years old^7,9^) suggested that increasing the difficulty of the task (e.g., by a decrease of the font readability in a reading task or presenting more complex math problems) would shield the listeners’ performance (both accuracy and RTs) against the presence of background noise. However, all the previous studies compared a noisy condition against a quiet one whereas in the present case background noise was always played back during the task, though at different levels. The present results hint at the possibility that the trade-off between task difficulty and listening condition could not be observed when, as is usually the case in real classrooms, background noise is always present.

In line with the prediction for the second research question of the study, we found that statistical models including individual and task factors are able to explain a higher variance in the data compared to models including only the acoustical factor. Research on adults^44^ and older students^61^ already outlined the importance of listener factors for examining performance on complex tasks in noise and especially for understanding why some listeners are particularly vulnerable. All statistical models have a higher conditional R^2^ than a marginal R^2^, meaning that the random effects included in the model (participants) explained a higher proportion of variance compared to the fixed effects. The difference between the two parameters might be interpreted to mean that the variation between the participants is more substantial compared to the variation within them (related for instance to the listening condition), thus reinforcing the idea that individual differences have a crucial role in determining how children react to the classroom sound environment. It is worth mentioning the low conditional R-squared (32.1% and 20.5%) of the models for accuracy and RT. Even though the result is in line with the proportion of variance explained for similar models of accuracy in complex tasks^61^, it suggests that other individual factors, not included in the present study, could be more influential in explaining inter-individual variance. For instance, the new perspective introduced by research on indoor soundscape suggests a potential role of affective responses (emotions, mood) in determining the outcome of cognitive tasks^62,63^. Also, switching skills and mind-wandering propensity were found to influence children’s ratings of interference and annoyance from noise^32^ and might in turn affect accuracy and effort.

It should be noted, however, that also the experimental setting could have influenced our findings. We chose to present the comprehension task in an ecological setting (a classroom with multiple distracters, with peers and a teacher present, and using a class-wise paradigm), thus differing from previous studies on the same topic using a more controlled testing situation (a laboratory, with limited distractors and a single child paradigm). As an example, children’s performance might be influenced by group dynamics, or by the presence of visual or unpredictable auditory distracters during the task. Despite these critical aspects, presenting experiments in the classroom has proved to be a more valid method of indexing cognitive abilities as predictors of academic attainments compared to the laboratory^43^. Studies in real-world classrooms^21,64^ or real complex acoustic scenarios^65^ are increasingly called for, to bridge the gap between the laboratory and the field, and derive recommendations and acoustic guidelines meeting as closely as possible the needs of children during learning.

Concerning the third research question pertaining students’ motivation, from the FUEL model^28^ activities are prioritized for resource allocation depending on both individual motivation and cognitive demands. That is, if a student has little motivation to understand the teacher’s message, a decrease in task demands (e.g., by reducing the classroom noise level) might result in little or no change in her/his effort anyway. The motivational consequences of chronic exposure to noise have been investigated in past research^66^ suggesting that children from noisy classrooms are less likely to persist when required to perform a challenging task and tend to give up on it more easily. The factors that motivate students to listen in the classroom are still not fully explored^67,68^. While roles have been suggested for the pleasantness of the teacher’s voice^26^, reverberation time^69^, and overall unstimulating and non-arousing classroom features^70^, no systematic assessment have been conducted. Our results suggest a role for literacy skills in children’s motivation. In particular, decreasing the noise level in the classroom will increase the motivation only for children with a better mastery of reading comprehension. It might be speculated that this higher level of motivation could help them to continue listening and not disengage from the lesson even when other factors (e.g., task complexity) will increase the cognitive demands. On the contrary, having fewer literacy skills resulted in no changes in motivation even when the sound environment was improved. Notably, in effortful listening, unless sufficient motivation can be raised, after a while the listener will stop concentrating as fatigue sets in^28^. Therefore, teaching/classroom management strategies in addition to background noise control should be applied for these students. It is worth highlighting here that the concept of bolstering students’ motivation to improve performance in the classroom is well-known in educational models^67^ and that research in this area would greatly benefit from a multi-disciplinary perspective.

Our study has practical implications for the design of inclusive learning spaces. By comprehending the interaction between individual characteristics and the classroom acoustic environment on students’ performance, effort, and motivation, we can better identify the most vulnerable students and provide the class with proactive solutions. School screening could be designed to include tests aimed at evaluating the most relevant individual factors, so as to identify the students who are most susceptible and address their needs. The findings of the study indicate that reducing noise generated by children might be beneficial for vulnerable peers. This reduction can be obtained through a design strategy or a pedagogical strategy (or a combination of both). The design strategy involves the acoustic treatment of the classroom, which has proved to be effective in reducing the levels of students’ activity noise at all levels of education^71–74^. The pedagogical strategy relies on the fact that children are the main source of noise, so noise levels can be managed by raising the children’s awareness of noise^75^, and/or developing noise-control behaviors^76^. This latter strategy could also help in attenuating the negative effects of noise on children’s motivation, as the lack of controllability of an environmental stressor was shown to be a critical factor in decreasing motivation^66^.

In summary, our results consistently indicated that a two-talker masker impairs children’s accuracy, effort, and motivation during listening comprehension in real classrooms. Its specific impact depended on the level and was moderated by the child’s individual characteristics. The increased proportion of variance explained by models that consider individual factors in addition to listening conditions, underscores the significance of considering individual aspects when assessing the influence of classroom acoustics on students, to design inclusive spaces promoting students’ health and well-being, and supporting their learning performance.

This study has several limitations that could be addressed in future research on this topic. First, we explored the moderation effect of individual factors on two measures of listening effort (RT and self-ratings). These measures were chosen over other behavioral and physiological measures because they were easier to be accomplished when testing in a real classroom^77^. Future studies should explore the association with other measures of effort, still considering the methodological constraints related to collecting physiological measures in a classroom setting. Second, our study did not directly investigate motivation as an explanatory variable for listening effort: rather it aimed at a preliminary clarification of the relationship between children’s individual factors, listening conditions, and motivation in noisy conditions. Third, noise sensitivity was assessed by using a test validated for the adult population, which might pose some validity concerns when used with children. Whereas no specific tests for children were available at the time of our experiment, future studies with primary school children could assess this internal state by using a recently-published questionnaire validated for primary school students^52^. Finally, the validity of the present results is limited to the specific task and listening condition chosen for the study. Further research is called for to explore the role of individual factors in other areas of learning (e.g., maths), different background noises (e.g., more competing talkers, unintelligible babble noise), and teaching methods (e.g., collaborative work instead of a lecture-style method).

## Methods

### Participants

Children between the ages of 8 and 11 were recruited from eight primary school classes in Ferrara, Italy (equivalent to Grade 3–Grade 5 in the US). The number of children in each class ranged between 12 and 18. Data from five children were removed from the analysis, due to the presence of certified hearing disorders (n = 1) or non-neurotypical development (n = 4). The final sample of participants included 104 students (Grade 3: n = 22, M = 8.3 years, SD = 0.5 years, 14 female; Grade 4: n = 55, M = 9.6 years, SD = 0.5 years, 32 female; Grade 5: n = 27, M = 10.9 years, SD = 0.3 years, 12 female).

The study was approved by the Institutional Review Board of Psychology of the University of Chieti-Pescara (Department of Psychological, Health and Territorial Sciences; Protocol number 22015) and was performed in compliance with the Declaration of Helsinki guidelines. Written informed consent was also obtained from the children’s parents.

### Design and procedure

A repeated-measures design was used in the study, with all children performing the comprehension task in two listening conditions. Children took part in the experiment as a whole class, and the tasks were administered collectively in the classroom in which they usually have lessons, with children seated at their desks. Each class completed the experimental task and a reading comprehension assessment in one session and an inhibition task and a noise sensitivity questionnaire in a second session, 1 week later. The order of the listening conditions in the sentence comprehension task was counterbalanced across the students of each class to avoid fatigue or practice effects on the task results. The absence of significant order effects on task results was confirmed by setting up statistical models including the order of presentation as an explanatory factor (for all the models *p*s > 0.46).

All tasks were programmed with an online testing platform (https://gorilla.sc/) and presented to the children using tablets. Sound stimuli were delivered via headphones (Sony MDR-ZX310) that were equalized with a two-step procedure based on (1) measurements of their frequency response with a B&K 4100 head-and-torso simulator, and (2) calculation of digital inverse filters to be applied to the input audio signals.

### Individual measures

#### Reading comprehension (literacy skill)

Comprehension of a narrative passage was assessed for all children, on the account that proficiency in understanding complex information can modulate the cognitive load of the task (i.e., a comprehension task might do fewer demands on children’s cognitive resources when they are more fluent and proficient in the task itself).

An adapted task based on comprehension of short passages^78^ was presented in quiet conditions on the same day as the experimental task. The task consisted of four short narrative passages, differing in subject content, length (46–56 words), and syntactic complexity (simple, complex). After reading a passage on the tablet, the participants had to answer four multiple-choice questions about it (two factual and two inferential questions). Each question with a correct answer was scored as “one” whereas a wrong/missing answer was scored as “zero”. Hence, the task had a maximum score of 16. The children’s scores ranged between 6 and 16, with a mean value of 11.6 (SD 2.2) Based on the median score of the whole sample (MD 12), participants were sorted into two groups (“low” and “high” reading comprehension).

#### Inhibition (cognitive skill)

Inhibitory control of attention was assessed concerning the auditory domain, by presenting stimuli over headphones as a go/no-go task (adapted from^79^). Stimuli consisted of pure tones presented binaurally at 60 dB: the first stimulus (S1) was a pure tone at 1000 Hz presented for 100 ms, and the second stimulus (S2) was obtained as a combination of two tones (tone at 1000 Hz presented for 100 ms—silence for 50 ms—tone at 800 Hz for 100 ms). S1 was presented on 70% of the trials and required response execution (button press response on the tablet—Go). S2 was presented in 30% of the trials and required inhibition of the response (not pressing the button—No Go). The inter-stimuli interval was set at 2000 ms. There were two blocks, each consisting of 50 trials, preceded by a 10-trial practice block.

It was explained to the children that they would hear a sound, either a tone that meant “press the button” or a tone that meant “don’t press the button”. During instructions both speed and accuracy in doing the task were required to them. Only presses to the Go tone with a response time shorter than 2000 ms were considered correct, although all button presses were recorded to monitor omission and commission errors. For each trial, accuracy (correct/wrong answer) and response speed were recorded. The number of commission errors (over a maximum number of 30) was used as a measure of inhibition and used to sort the participants into two groups (“low” and “high” inhibition, based on the median split of the distribution).

#### Noise sensitivity

Noise sensitivity was measured using the reduced Italian version of the Weinstein Noise Sensitivity Scale^80^. The children had to indicate their agreement on five statements related to their sensitivity to noise (e.g., “I find it hard to relax in a place that’s noisy”). For each statement, the level of agreement could be chosen on a 5-point scale (from 1 “not at all” to 5 “very much”).

To derive an overall score, the score of the last statement was flipped to match the direction of the others (i.e., with higher scores implying a higher sensitivity to noise) and then the mean value over the five statements was calculated. The overall scores ranged between 1 and 5, with a mean value of 3.1 (SD 0.8). Participants were then sorted into two groups (“low” and “high” noise sensitivity) based on the median score of the sample (MD 3.1).

### Testing measures

#### Sentence comprehension task

The sentence comprehension task was designed to assess the children’s ability to listen and comprehend a sentence in the presence of competing background noise. The task was specifically designed for this study by adapting materials from a standardized sentence-to-picture test developed for Italian (Comprendo^81^). It is based on a sentence-picture matching tests that requires the listener not only to segregate the target speech from the noise (speech perception), but also to perform higher-level cognitive processes including linguistic and syntactic parsing (comprehension).

**Figure 4:**
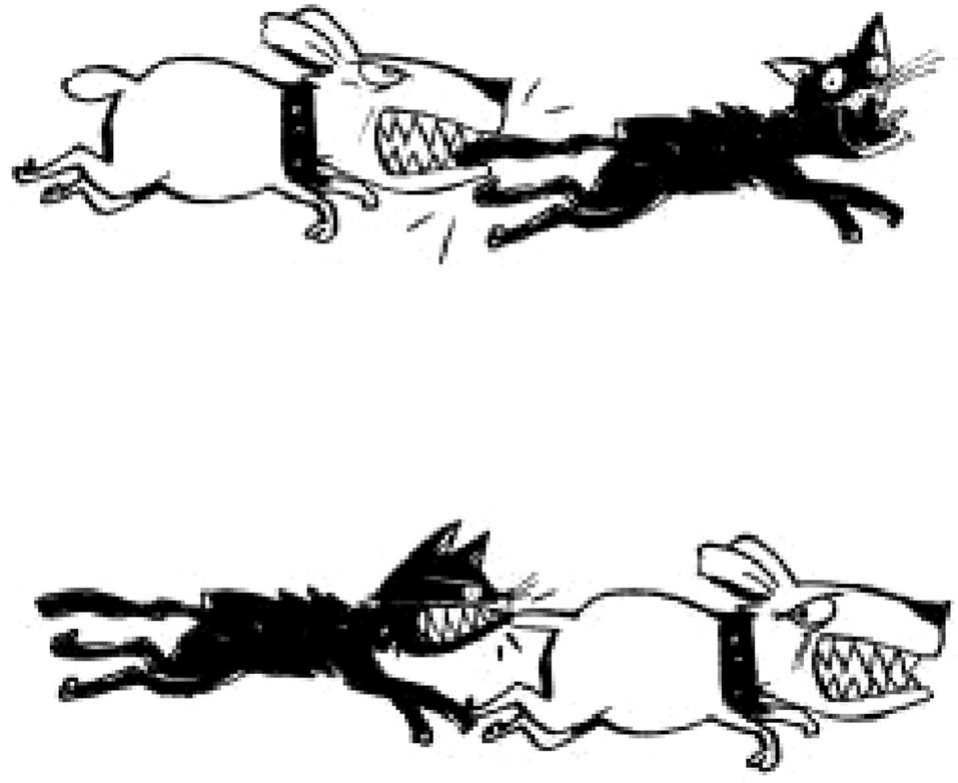
Example of a set of images in the comprehension task. The target sentence was an object relative: “Point to the cat that the dog bites”.

For each listening condition, 15 sentences were presented to the children. The sentences were split into three blocks, in which they were counterbalanced by syntactic complexity (low complexity: coordinate, passive objective relative, passive; high complexity: clitic, relative objective). For each trial, participants listened to the playback of a sentence, with the noise starting around one second before the speech onset and ending at the same time. At the audio offset, two images appeared on the tablet and they had to select the image that best matched the sentence content (Fig. 4). It is worth noticing that in the image pair the competitor picture showed the same characters as the target picture, but with the roles inverted. That would require the children to mentally assign the roles (“who is doing what to whom”) in order to complete the comprehension task. The image selection task was time-limited to 15 s.

Accuracy and response time (RT, the time elapsed between the end of the audio playback and the moment an answer was selected) were recorded for each sentence.

#### Self-reported listening effort and motivation

Self-reported effort and motivation in completing the task were measured on visual analog scales at the end of each listening condition. The following questions were formulated: “How hard did you have to work to understand the previous sentences?” (after^82^) and “How important was it to you to perform well in the task?” (after^30^). It is worth mentioning that studies on subjective ratings of effort for school-aged children are still scarce and no consensus on the appropriate wording of the questions has been reached. However, all studies consistently point out a discrepancy in the results of behavioral, physiological, and subjective measures of effort (see, for instance^83^). Participants responded to the questions using a slider bar with values ranging from 0 to 100 in increments of 1. The slider was initially positioned on the midpoint of the scale. Verbal anchors (“Not at all”, “Extremely”) were positioned at each endpoint of the slider bar.

### Listening conditions

The sentence comprehension task was performed in two listening conditions, simulating the sound environment of a classroom with a volume of 256 m^3^, and a reverberation time at the medium frequencies equal to 0.73 s (complying, also in the octave-band distribution, with the Italian acoustic standard UNI 11532-2). The classroom was simulated in the room acoustic modelling program ODEON. A virtual listener was positioned in the centre of the area where students usually sit, with the talkers surrounding it, at nearly 1.5 m distance (front-right and back-left). A third speech source was simulated at the teacher position, close to the front of the classroom, in line with the receiver. The binaural impulse responses simulated in the virtual classroom were then convolved with the anechoic recordings of two primary school girls reading aloud passages from different age-appropriate books and a female speaker reading the target material of the comprehension task.

For all the listening conditions of the experiment, the target speech level was set to 60 dB(A). The two listening conditions were obtained by varying the two-talker noise level from 59 to 51 dB(A), to obtain SNRs equal to + 1 and + 9 dB (“hard” and “easy” listening conditions, respectively). Both SNRs fit within the range of values measured in actual classrooms^84^, with the hard condition chosen to be at the more challenging end to increase the involvement of cognitive resources.

### Statistical analyses

Data analysis was performed using *R* (v4.1.0), *RStudio* (v2022.07.2), and the *afex* package (v1.2-0 ^85^,) for all mixed-effects model analysis.

For all mixed models, first the *maximal model* (i.e., the “maximal random effect structure justified by the design”^86^) was defined. Because all factors in the experiment varied within-subjects, the maximum random effect structure involved by-participant random intercepts as well as by-participants random slopes for the factors listening condition and task complexity, and their interaction. Moreover, the by-participant random intercept was nested within class and school. The by-item random intercept was included as well, to model differences that might exist between the sentences of the experimental task^87^. Then, due to convergence warnings, the maximal random effect structure was reduced, by removing interactions among the random terms, the random slope for the factor task complexity, and the nested structure for the by-participant intercept. The converging model with the simplified random effects structure and all the effects of interest as fixed factors was set up and fitted by using the function *afex::mixed*. Likelihood ratio tests (*method* = *“LRT”*) were used to test all fixed effects in the model. The *p*-values and χ^2^ values for the models were obtained with the *afex* package.

Accuracy data were analysed with a binomial generalized linear mixed-effects model (GLMM), as data were categorized as either 0 (wrong response) or 1 (correct response). Response time data were analysed with a GLMM using a distributional Gamma family with a logarithmic link function, given the right-skewed distribution of the data. Self-reported effort and motivation were analyzed using linear mixed-effects models. Post-hoc tests were performed with the *emmeans* package (v1.8.2,^88^). In the case of multiple comparisons, the *p*-values were adjusted using the Bonferroni procedure.

In order to answer the first research question a “full” model was set up for each measure of the study. This “full” model included the simplified random structure and the following fixed effects: listening condition (easy/hard), reading comprehension (low/high), inhibition (low/high), and noise sensitivity (low/high). The two-way interactions between the listening condition and the individual factors were included in the models, to investigate how individual characteristics interact with the effect of the listening condition. The GLMMs for accuracy and RT included the fixed effect of task complexity (low/high) as well. In addition, a “base” model was set up for each measure of the study, having the same random structure as the “full” model, and inlcuding only the listening condition as a fixed effect. Marginal and conditional coefficients of determination (R^2^_m_ and R^2^_c_) were calculated for each “full” and “base” model using the package *performance* (v0.9.2,^89^). The R script used for the analysis of accuracy data is reported as Supplementary material online, as an example of the analytical strategy adopted in the study.

### Supplementary Information

Supplementary Information.

## Data Availability

The data that support the findings of this study are available from the corresponding author, CV, upon request.
