# Supplementary material for: Individual characteristics moderate listening effort in noisy classrooms
Source: Sci Rep. 2023 Aug 31;13:14285. doi: 10.1038/s41598-023-40660-1 (PMC10471719; doi:10.1038/s41598-023-40660-1)
Supplement: Supplementary file 1 — Supplementary Information. [file 41598_2023_40660_MOESM1_ESM.pdf]

SUPPLEMENTARY MATERIAL – R code

This Supplementary Material reports the R script used for the statistical analysis of the dataset used in the manuscript "Individual characteristics moderate listening effort in noisy classrooms" (Visentin et al., *Sci Rep*, 2023)

The same analytical strategy was adopted for the analysis of response time data and subjective ratings.

```
#### load libraries

library(lme4)
library(afex)
library(dplyr)
library(tidyr)
library(sjPlot)
library(performance)
library(emmeans)
library(ggplot2)

###import the dataset for accuracy data

library(rstudioapi)
current_path = rstudioapi::getActiveDocumentContext()$path
setwd(dirname(current_path ))

load("Data_schools.RData")

### data cleaning

# remove timeouts (>15 s)
all_data=subset(all_data,!Timeout=="1")

# remove trials with RT<150 ms
all_data =subset(all_data,!RT<150)

# remove participants without individual measures
sub_data = all_data %>%
  drop_na(noise_sensitivity) %>%
  drop_na(reading_comprehension) %>%
  drop_na(inhibition)

### recoding levels for each variable
sub_data$noise_sensitivity <- relevel(factor(sub_data$noise_sensitivity), ref="Low")
sub_data$ reading_comprehension <- relevel(factor(sub_ data $ reading_comprehension), ref="Low ")
sub_data$inhibition <- relevel(factor(sub_ data $ inhibition), ref="Low")
sub_data$list_cond <- relevel(factor(sub_ data $ list_cond), ref="Hard")
sub_data$complexity <- relevel(factor(sub_ data $complexity), ref="Low")
```

statistical model

```
# maximal model (not converging)
mod<-mixed(correct ~ list_cond + complexity + noise_sensitivity + inhibition + reading_comprehension +
           inhibition + list_cond: noise_sensitivity + list_cond: reading_comprehension + list_cond:inhibition+
           (list_cond*complexity |school\class\participant)+(1|item),
           data=sub_data, family=binomial,method='LRT')

# full model (no warnings)
mod<-mixed(correct ~ list_cond + complexity + noise_sensitivity + inhibition + reading_comprehension +
           inhibition + list_cond: noise_sensitivity + list_cond: reading_comprehension +  list_cond:inhibition+
           (list_cond |participant)+(1|item),
           data=sub_data, family=binomial, method='LRT')

# base model
mod.b<-mixed(correct ~ list_cond +
          (list_cond |participant)+(1|item),
         data=sub_ data, family=binomial, method='LRT')

# get coefficients of determination
r2_nakagawa(mod)
r2_nakagawa(mod.b)

#post-hoc
e<-emmeans(mod, "complexity", type = "response")
pairs(e, adjust = "bonferroni")
```
